# Abuse of the Pessary

**Published:** 1858-06

**Authors:** J. S. Weatherly

**Affiliations:** Montgomery, Alabama


### ARTICLE II.

*Abuse of the Pessary.* By J. S. WEATHERLY, M. D. Montgomery, Alabama.

Much has been said recently against the abuse of the speculum; and as it seems almost impossible for the profession to get hold of anything without abusing it, I ask your indulgence for a short time, whilst I draw forth another instrument, whose use, I think, has been more abused, and been productive of more injury than the speculum. I refer to the pessary —an instrument coming down to us through the long vista of years, with the habiliments of the ancients clinging around it —sanctified even by the hand of the father of medicine himself, who was said to have used some fourteen different kinds of pessaries. But his idea was not, however, so much the curing of prolapsion, and kindred diseases, by mechanical means, as by medication; for I believe all of his instruments were medicated.

Modern physicians have almost entirely discarded the medicated pessary, (probably without sufficient reason,) and use them merely as a mechanical support; though not a few authors refer to them as curative agents, in the treatment of prolapsion. Meigs devotes some three or four of his letters to this subject. But with all due deference, and high respect for his talents, we must say that three or four pages would have done as well, and made the author more reputation than his long dialogue with Miss H. Blanque. He is, however, very sensible on the subject, when he gets to it, (as he generally is when he gets to the point.) He points out some of the bad effects of this instrument, when it is unsuited to the case, such as ulceration of the vaginal canal, recto vaginal fistula, &c. He also says, that the globe pessary, which he prefers, will frequently not be retained, on account of the relaxation of the vaginal canal, and that recourse must be had to the stem pessary, which it seems to me must necessarily produce more injury from its mechanical irritation, than any advantages that could possibly be derived from its employment.—That they also are well calculated to produce leucorrhœa, ulceration, etc., there can be no doubt. The simple globe pessary, however, it seems to me is less liable to these objections, than any of the others. In fact, in the majority of cases, where they are used, these things already exist, either as effect or cause, and it matters not which, so far as the pessary is concerned, but so far from the pessary curing the prolapsion, if such really exist, it aggravates all of the symptoms.

It does not require very keen conception, it seems to me, to see that any foreign body, constantly in contact with the mucus membrane of the vagina, and the os uteri, is almost necessary compelled to produce changes in the parts, even if they are clear of disease previous to its introduction. And especially is this the case, if there be predisposition to inflammation of the mucous membrane. For instance, a patient applies for medical assistance. She informs you that she has an uneasy feeling in the lower part of her bowels, with pains in her back and hips, and winds up by saying that she thinks her womb is misplaced. (The majority of the sex think that all diseases of the womb consist in a displacement, and in this they have the company of some learned M. D.'s also.) You examine the case, and if imbued with the idea that all uterine diseases are caused from some malposition of the organ, you very readily make out a case of prolapsion. Why? From the fact that it probably does exist to a greater or less extent, as a symptom of some other disease. But if you push your enquiries a little farther, and use your reason as well as your fingers, you will probably arrive at a more correct diagnosis of your case. Even with the fingers you can feel that the uterus is heavier than natural, that the mouth is dilated, and that the lips are puffy and soft, or hard and painful; also, that the vagina is tender, &c. And if you now introduce that HORRID *speculum*, you will perceive the mouth of the uterus looking as rosy as a full ripe strawberry, and probably covered with minute points of ulceration. But suppose, instead of the speculum, you introduce the more elegant pessary, (for it does seem to me that the one is about as indelicate as the other;) do you cure your patient? Most certainly not; but on the contrary, all the symptoms will be aggravated, and you will be forced to remove the instrument. And it matters not what kind of instrument you use—whether globe, disc or stem—all are alike objectionable.

Though the pessary, in some few cases, is no doubt a valuable adjunct, in the treatment of prolapsion, I doubt very much whether it ever cures a case. When I first commenced the practice of my profession, I thought I had, or would have, frequent use for this little instrument. Consequently I procured a fine assortment; and, as a matter of course, I used them as often as I thought a case would admit of one, and probably much oftener than was really necessary. But after studying more particularly the nature of uterine and vaginal diseases, I found that their use was rarely indicated, and that I could treat the majority of cases much more satisfactorily to myself and patients without them, for I can't conceive of any thing more annoying to a female, than to have one of these instruments constantly in her vagina. Again, I found that it was almost impossible to keep one of them adjusted in the vagina of a negro woman. Their duties are such, generally, as to make it very difficult, even if they tried, to retain them in situ.

Among the better class of females, particularly our Southern ladies, whose sedentary habits are constantly predisposing them to uterine derangements, we find prolapsion, when it does exist, more as a symptom, than as a disease, *per se.* And we can cure it by remedying the engorgement, or ulceration, if it exist; not, however, by introducing a pessary, and sending our patients to bed, but by using rational means for subdueing the disease, and giving tone to the relaxed tissues.

